# Engineering of Human Skeletal Muscle With an Autologous Deposited Extracellular Matrix

**DOI:** 10.3389/fphys.2018.01076

**Published:** 2018-08-20

**Authors:** Lieven Thorrez, Katherine DiSano, Janet Shansky, Herman Vandenburgh

**Affiliations:** ^1^Tissue Engineering Laboratory, Department of Development and Regeneration, KU Leuven Kulak, Kortrijk, Belgium; ^2^School of Medicine, Case Western Reserve University, Cleveland, OH, United States; ^3^Department of Pathology, The Miriam Hospital, Brown University, Providence, RI, United States

**Keywords:** tissue engineering, skeletal muscle, collagen, fibrin, extracellular matrix, viscoelastic properties

## Abstract

Adult skeletal muscle progenitor cells can be embedded in an extracellular matrix (ECM) and tissue-engineered to form bio-artificial muscles (BAMs), composed of aligned post-mitotic myofibers. The ECM proteins which have been used most commonly are collagen type I and fibrin. Fibrin allows for *in vitro* vasculogenesis, however, high concentrations of fibrinolysis inhibitors are needed to inhibit degradation of the ECM and subsequent loss of BAM tissue structure. For *in vivo* implantation, fibrinolysis inhibition may prove difficult or even harmful to the host. Therefore, we adapted *in vitro* culture conditions to enhance the deposition of *de novo* synthesized collagen type I gradually replacing the degrading fibrin ECM. The *in vitro* viscoelastic properties of the fibrin BAMs and deposition of collagen were characterized. BAMs engineered with the addition of proline, hydroxyproline, and ascorbic acid in the tissue culture medium had a twofold increase in Young’s Modulus, a 2.5-fold decrease in maximum strain, and a 1.6-fold increase in collagen deposition. Lowering the fibrin content of the BAMs also increased Young’s Modulus, decreased maximum strain, and increased collagen deposition. Tissue engineering of BAMs with autologous ECM may allow for prolonged *in vivo* survival.

## Introduction

Adult skeletal muscle progenitor cells can be engineered *ex vivo* into contractile tissues containing post-mitotic myofibers (Vandenburgh and Kaufman, 1979). To obtain 3D tissues, proliferating human myogenic cells are embedded in ECM hydrogels and then differentiated to form organized myofibers. Myofiber alignment occurs due to the uniaxial passive forces generated in the gel when it contracts away from its casting mold containing two attachment sites. The resulting engineered muscle bundles are termed as bio-artifical muscles (BAMs). BAMs were demonstrated to provide insights in drug screening (Vandenburgh et al., 2008, 2009; Vandenburgh, 2010) and disease modeling (Chromiak and Vandenburgh, 1992; Vandenburgh et al., 2009; Lee and Vandenburgh, 2013). Ultimately, BAMs may provide a replacement for damaged or atrophied muscle tissue (Powell C.A. et al., 2002). In addition, the progenitor cells can be genetically engineered to secrete therapeutic proteins such as growth hormone, VEGF, and blood clotting factor IX. When these cells are then used to create BAMs which are implanted, they can serve as an *in vivo* protein delivery system (Vandenburgh et al., 1991, 1996, 1998; Lu et al., 2001, 2002; Shansky et al., 2006a; Thorrez et al., 2006). BAMs with post-mitotic muscle fibers have a better safety profile than injecting individual proliferating and migrating cells in the host because of reversibility of implants in case of an adverse event (Vandenburgh et al., 1998; Lu et al., 2001, 2002; Thorrez et al., 2006). *Ex vivo* differentiated myofibers exhibit improved cell survival kinetics when compared to implanted myoblasts, with a gradual loss of viability over 30–60 days after implantation (Vandenburgh et al., 1996; Thorrez et al., 2006). The latter may be partially due to poor myofiber tension maintenance *in vivo* (Thorrez et al., 2006). Seeding cells on a biodegradable scaffold is an approach which may provide temporary mechanical strength to the tissue and help maintain myofiber tension. However, with scaffolds, myofiber alignment remains a critical issue and scaffold degradation products may affect myofiber survival (Thorrez et al., 2008). Successful transplantation of skeletal muscle cells (either myoblasts or myofibers) for structural repair or *ex vivo* gene therapy applications thus requires further optimization for long term cell survival.

In most of the work we published up to date, type I collagen has been used to form the hydrogel, motivated by the fact that it is the most prominent ECM protein in adult skeletal muscle (Vandenburgh, 1988). Initially, rat tail collagen I was used (Vandenburgh et al., 1988), but with the aim of developing a clinical product, clinically approved collagen type I (Zyderm) was used (Thorrez et al., 2006). We found however, that Zyderm strongly inhibits vascularization of the BAMs *in vivo* (Thorrez et al., 2006). Vascularization of the BAMs is desirable to allow for an efficient delivery of nutrients to the myofibers and offloading of the therapeutic protein. Even secretion of high levels of VEGF from Zyderm BAMs is unable to overcome this anti-angiogenic effect (Thorrez et al., 2006). Therefore, an alternative clinically approved ECM protein was needed to allow vascularization of BAMs.

Fibrin is commercially available (e.g., Tisseel) and is used widespread in clinical applications as a tissue glue. It is also used as an ECM in many other bioengineering applications due to its structural and mechanical properties (Helgerson et al., 2004). The structural properties of the fibrin gel can be varied by manipulating a number of parameters, such as the fibrinogen and thrombin concentration, additional crosslinking, and ion concentration (Helgerson et al., 2004). Fibrin extracellular matrices are subject to degradation *in vitro* and *in vivo* via fibrinolysis. This can be inhibited *in vitro* by adding the enzymatic inhibitors aprotinin and/or tranexamic acid to the culture medium. Although this prevents fibrin degradation *in vitro*, once fibrin is used *in vivo*, it is subject again to fibrinolysis. In previous studies, when fibrin was used as an ECM for tissue engineering, the fibrin was reabsorbed within 2 weeks (Beier et al., 2006).

In this paper, we present an adapted *in vitro* engineering procedure to improve viscoelastic properties of BAMs and circumvent BAM degradation due to proteolysis of fibrin. Deposition of autologous collagen by (myo)fibroblasts present in the BAMs may be induced by adding supplements to the culture media *in vitro*. Functional collagen contains a large amount of amino acids that have been post-translationally modified, specifically proline to hydroxyproline. The conversion of proline to hydroxyproline is catalyzed by the enzyme prolyl 4-hydroxylase which requires ascorbic acid (vitamin C) for its enzymatic function (Nelson and Cox, 2005). Proline and hydroxyproline constitute roughly 30% of the amino acids in collagen, stabilize the helix and increase the amount of hydrogen bonds (Nelson and Cox, 2005). Experiments were performed in which the effect of proline, hydroxyproline, and ascorbic acid as media supplements were evaluated to determine effects on autologous collagen deposition in fibrin BAMs and changes related to viscoelastic characteristics. We investigate the effects of this medium supplementation together with other parameters including fibrin density, fibrin crosslinking, and cell density.

## Materials and Methods

### Muscle Progenitor Cell Isolation and Culture

Primary human skeletal muscle cells were isolated by needle biopsy of the vastus lateralis from healthy adult volunteers (Powell et al., 1999; Powell C.A. et al., 2002; Shansky et al., 2006b) and cells were 70% myogenic on average as determined by desmin staining (Powell C.A. et al., 2002). Biopsy and isolation protocols have been previously described in detail (Powell et al., 1999; Powell C.A. et al., 2002; Powell C. et al., 2002; Shansky et al., 2006b) and were approved by the Institution Clinical Review Board of the Miriam Hospital. Human skeletal myoblasts were cultured in human SkGM, SkGM/15 [SkGM Bullet Kit (Cambrex Bio Science, Walkersville, MD, United States) + 15% (v/v) FBS]. Cells were cultured in a 37°C, 5% CO_2_ incubator and media was changed every other day.

### Engineering of Fibrin BAMs

BAMs 20 mm in length were engineered in an elliptical, shallow-welled silicone mold affixed to the bottom of a standard six-well tissue culture plate. Two stainless steel pins (1 mm diameter) were placed into the mold 20 mm apart and served as an attachment site. Custom-made transfer devices (for transfer to the viscoelastic testing device) made of stainless steel cylinder (inner diameter 0.038 in) bent in a U-shape were inserted over the pins. Two million skeletal myoblasts were used for each BAM. Cells were mixed with fibrin ECM [0.25–4 mg/ml fibrinogen + 0.5–2 U/ml thrombin (Tisseel, Baxter)] in a total volume of 500 μl. In some experiments FXIII (0.4 U/ml) was added. Cell-ECM mixes were incubated at 37°C and 5% CO_2_ for 2 h followed by addition of 8–10 ml SKGM/15 containing 1,000 U/ml aprotinin (Trasylol^TM^). Where indicated, the following media supplements were added: 40 mg/L L-proline (Sigma), 10 mg/l trans-4-hydroxy-L-proline (Sigma), and 0.1 mM L-ascorbic acid 2-phosphate (Sigma). The solidified cell-ECM was detached from the silicone mold using a sterile fine dentistry tool (FST). This enabled the cells to contract around the pins and form unidirectional, aligned myofibers.

After 2 days, myoblast fusion into post-mitotic myofibers was induced by incubation in differentiation medium [high glucose DMEM (Gibco), 50 μg/ml bovine serum albumin (Cambrex), 50 μg/ml gentamicin (Sigma), 10 ng/ml epidermal growth factor (Cambrex), and 10 μg/ml insulin (Cambrex)] with 1,000 U/ml aprotinin and with or without supplements. For some BAMs, aprotinin was stopped at day 5. Medium was changed every 2–3 days and BAMs were kept at 37°C, 5% CO_2_. BAMs were maintained in culture for 7–14 days before viscoelastic testing.

### Collagen (Zyderm) Control BAMs

Collagen BAMs were engineered in a manner previously described (Powell et al., 1999; Powell C. et al., 2002; Powell C.A. et al., 2002; Shansky et al., 2006b; Thorrez et al., 2006; Decroix et al., 2015). Briefly, 2 million human myoblasts were mixed in 1ml of a 1 mg/ml collagen type I ECM (Zyderm^TM^, Inamed) and treated similar to the fibrin BAMs. Manual detachment from the mold was not needed since the collagen ECM does not adhere to the silicone mold as much as the fibrin ECM.

### Viscoelastic Testing of BAMs

**FIGURE 1:**
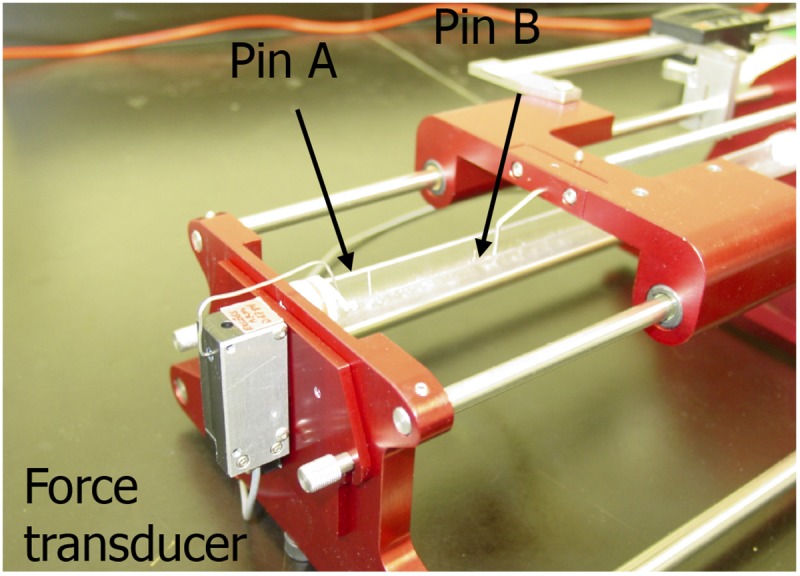
Image of the custom made visco-elastic testing device. Pin A is connected to the force transducer and pin B is connected to a stepper motor. Data were collected with a Labview virtual instrument.

Before testing, BAMs were imaged and center diameter was measured using ScopePhoto software. The viscoelastic testing of the BAMs was performed using a custom-made device (**Figure 1**), consisting of a force transducer and a stepper motor connected to two different pins. Hardware was controlled by a virtual instrument designed in Labview software (National Instrument). The two pins are set within a fluid cylinder filled with PBS at 36–37°C. BAMs were stretched at 0.9 cm/min until 2,500 μN.

Recorded data were imported into Microsoft Excel. Stress was calculated by dividing force by cross sectional area. A stress–strain curve was created, and the slope of the linear region of the curve was recorded as Young’s Modulus.

### Force Generation

BAMs were electrically stimulated via two platinum electrodes 4 mm apart on either side of the BAM. The voltage and stimulus were controlled using a custom designed LabVIEW program. Maximum isometric tetanic force was obtained by applying an electrical stimulus of 40 V at 40 Hz with 4 ms-wide pulses.

### Sprouting Assay

Sprouting assay was performed as described by Matsumoto et al. (2007). Briefly, dextran beads (Cytodex3, GE Healthcare, Piscataway, NJ, United States) were coated with HUVECs and resuspended at 800 beads/ml in fibrinogen solution (3 mg/ml) containing aprotinin (5 mg/ml). The resulting solution was mixed with a thrombin solution (2 U/mL) in a 5:4 ratio. Where indicated, either 0.1 or 1 mg/ml Zyderm was added to the fibrin. A total of 450 μL of this solution was then poured into each well of a 24-well plate and the plates placed in an incubator for 20 min. EBM-2 (Cambrex), which contains 2% FBS, was used as the medium for cell culture and where indicated, 10 ng/mL HGF (rhHGF, Chemicon) and 10 ng/mL VEGF were added. The ratio of beads containing sprouts, as a function of total bead number, was calculated after 6 days of culture using microscopic examination of the cultures.

### Histology

BAMs were fixed in 4% formaldehyde for 1 h and rinsed 3 × 5 min in PBS.

For tropomyosin staining, samples were permeabilized with methanol, incubated with mouse anti-tropomyosin antibody (Sigma #T9283) 1:100, followed by a secondary biotinylated anti-mouse IgG and then a preformed avidin and biotinylated horse radish peroxidase complex (Vector Laboratories, Burlingame, CA, United States). Development was by addition of DAB to produce a brown precipitate. Fixed BAMs were sent out for cross-sectional sectioning and hematoxylin and eosin staining (Mass Histology Service, Worcester, MA, United States). Slides were deparaffinized and then gradually rehydrated from 100% ethanol to H_2_O and stained for collagen using Accustain Trichrome Stains (Masson) (Sigma-Aldrich). The Trichome protocol was adapted as follows. Slides were soaked overnight in Bouin’s solution (Sigma), stained in hematoxylin (Sigma), and then rinsed in running tap water. The slides were then placed in Biebrich scarlet acid fuchsin (Sigma), followed by 12 min in a working phosphotungstic/phosphomolybdic acid solution (Sigma), and then 6 min in an aniline blue Solution (Sigma). Sections were rinsed in double distilled water in between every staining step. The slides were then destained for 1 min using a 1% acetic acid solution (Sigma), dehydrated with ethanol, and mounted in Polymount (Polysciences Inc.).

### Quantification of Deposited Collagen

A total of 12–15 non-overlapping images of each BAM were taken on a Zeiss Axiovert 25 microscope with a 40× objective using Scopephoto. The percentage of collagen (blue area) was quantified for each image with Metamorph Offline ver 6.3 and was recorded in Microsoft Excel.

### Statistics

Statistical analysis was performed using Instat (Graphpad) software. Non-parametric ANOVA tests (Kruskal–Wallis) with Dunn’s post tests were used. All data are presented as mean ± SEM and significance levels are indicated with ^∗^*P* < 0.05, ^∗∗^*P* < 0.01, and ^∗∗∗^*P* < 0.001.

## Results

### Sprouting Assay

**FIGURE 2:**
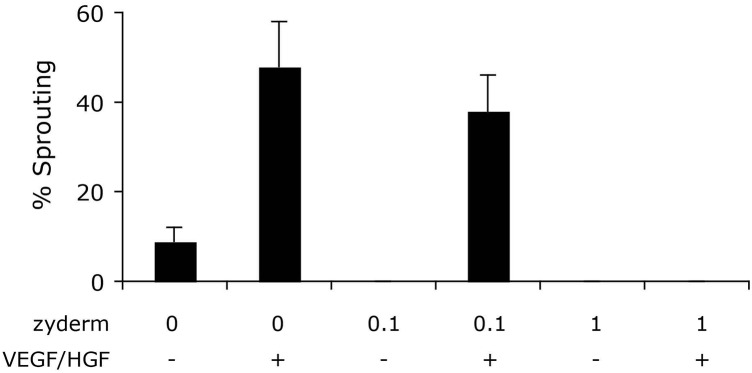
Sprouting assay. Different ECM compositions were tested *in vitro* for angiogenic sprouting. Fibrin was used as the standard ECM and was supplemented with 0, 0.1, or 1 mg/ml Zyderm. ECM compositions were incubated in the presence or absence of HGF + VEGF (both 10 ng/ml). The ratio of beads containing sprouts after 6 days of culture is displayed as a percentage of total bead number.

To compare different ECM compositions for angiogenic potential, we performed a bead sprouting assay. Standard ECM composition was fibrin, supplemented with no, 0.1 or 1 mg/ml collagen I (Zyderm^TM^). All ECM compositions were tested in the presence or absence of the angiogenic growth factors HGF and VEGF (10 ng/ml each). After 6 days of culture, sprouting beads were quantified relative to total beads (**Figure 2**). In the absence of Zyderm, 9 ± 3% sprouting beads were present, which increased to 48 ± 10% in the presence of growth factors. However, when Zyderm was added, no sprouting was observed without growth factors. Even in the presence of growth factors, 1 mg/ml Zyderm completely abolished sprouting. These data prompted us to search for engineering methods where the Zyderm collagen I could be omitted.

### Stimulation of Collagen Deposition

**FIGURE 3:**
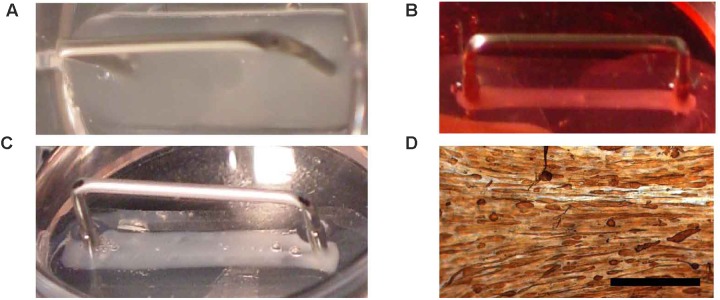
Fibrin BAM engineering. Fibrin BAMs are shown at different stages of engineering. **(A)** Cell-ECM mix in the silicon mold; metal part is a 2-cm long transfer device around which the cell-ECM mix contracts. **(B)** Two hours after casting, the BAMs were flooded with growth medium and the cell-ECM mix started to contract. **(C)** Two days later, BAMs are incubated in differentiation medium and contraction completes to form a 2-cm long, ∼1 mm thick tissue. **(D)** Whole mount tropomyosin staining of a human BAM shows that most myoblasts have fused to form myofibers aligned along the axis of the attachment sites. Scale bar is 500 μm.

As a substitution for the collagen I, fibrin was used as the ECM. Several conditions for engineering of fibrin BAMs were investigated. Since fibrin is degraded by fibrinolysis, we added aprotinin to the medium to inhibit fibrinolysis. Since fibrinolysis also occurs *in vivo*, we investigated a way to avoid construct degradation by fibrinolysis. Omission of aprotinin at day 5 of the engineering procedure allowed for gradual fibrinolysis during the remaining culture time. In a first series of experiments, we aimed at the enhancement of autologous collagen deposition to counteract the detrimental effect of fibrinolysis on tissue integrity and strength. To enhance autologous collagen deposition, culture medium was supplemented with ascorbic acid (0.1 mM), proline (40 mg/l), and hydroxyproline (10 mg/l). Similar to our previous work with collagen-based BAMs (Thorrez et al., 2006), BAMs contained 2 million human myoblasts. Different steps of the engineering process are depicted in **Figure 3**. Within minutes after combining the fibrinogen, thrombin and cells, the ECM gelled (**Figure 3A**). When media was added, the cell-ECM mix was manually detached from the silicon mold, allowing the cells to contract and attach solely to the pins (**Figure 3B**). Switching to differentiation medium at day 2 induced cell fusion and further contraction of the BAM (**Figure 3C**). The BAMs were maintained *in vitro* for 7–14 days before they underwent further characterization. Tropomyosin staining indicated that myoblasts had fused to form myofibers aligned in one direction (**Figure 3D**).

**FIGURE 4:**
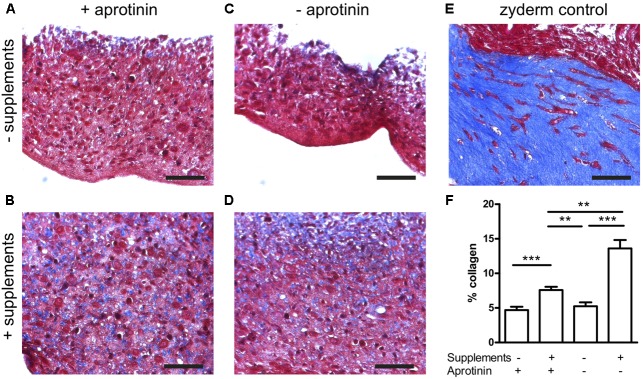
Collagen deposition induced by medium supplementation with ascorbic acid, proline, and hydroxyproline (+supplements) and in the absence (stopped at day 5) or presence of aprotinin. Images from Trichome stained sections from different BAM groups, *blue areas* are collagen staining. **(A–D)** Fibrin BAMs with 1 mg/ml fibrin, additions to the media as shown on the graph. **(E)** 1 mg/ml collagen (Zyderm) BAM as positive control for the collagen staining. Scale bars are 100 μm. **(F)** Depicts collagen quantification. ^∗∗^*P* < 0.01 and ^∗∗∗^*P* < 0.001.

Collagen deposited by the cells in the fibrin BAMs was visualized on histological sections with Masson’s Trichrome stain as a blue color (**Figure 4**). Collagen BAM sections were used as a positive control. In the standard condition without supplements and in the presence of aprotinin, 4.7 ± 0.5% collagen was deposited (**Figures 4A,F**). However, when supplements were added, this significantly (*P* < 0.001) increased 1.6 fold to 7.6 ± 0.5% (**Figures 4B,F**). When aprotinin was stopped without the supplements, the BAM integrity was severely affected by day 14 as the fibrin had started degrading and only 5.2 ± 0.6% collagen had been deposited (**Figures 4C,F**). When supplements had been given, then even in the absence of aprotinin (and thus in fibrinolytic conditions) degradation of the BAM structure was not visible and 13.6 ± 1.2% collagen had been deposited (**Figures 4D,F**). This was not only significantly higher (*P* < 0.001) than when not giving supplements, but also higher (*P* < 0.01) than the BAMs given supplements when aprotinin was also present. Both changes together, adding supplements and omitting aprotinin, resulted in a 2.9-fold increase of collagen content. In the absence of supplements, there was no significant difference in collagen deposition whether or not aprotinin was stopped on day 5.

**FIGURE 5:**
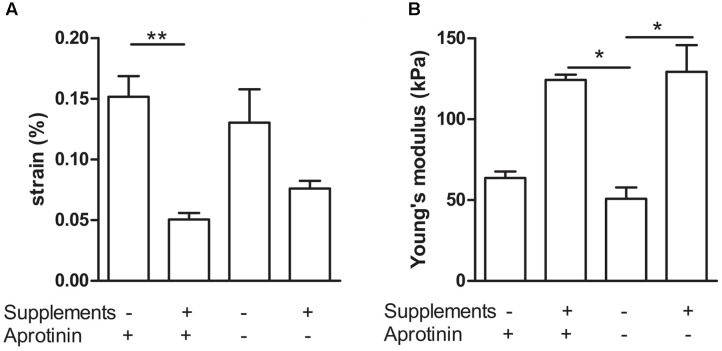
Visco-elastic effects of collagen deposition and fibrinolysis. Combination of addition (+) versus omission (–) of medium supplements and continued (+) versus discontinued (–) use of the fibrinolysis inhibitor aprotinin. **(A)** Strain of the BAM at 2,500 μN force. **(B)** Young’s modulus. Data are presented as mean ± SEM. ^∗^*P* < 0.05 and ^∗∗^*P* < 0.01.

To further quantify the effects of medium supplementation and induction of fibrinolysis, we determined visco-elastic parameters. With a custom made visco-elastic testing device (**Figure 1**), we mechanically stretched the BAMs and recorded stress–strain curves. Based on these curves, we measured the strain of the BAM when 2,500 μN force was applied and calculated Young’s modulus (*n* = 4–7 for each group). A low percentage (% of original length) of strain indicates that the BAM resists the applied force better. The stress–strain profiles display a non-linear toe region followed by a linear region (data not shown). The slope of this linear region of the forward curve corresponds to Young’s Modulus. We compared the effects of supplement addition and continued versus discontinued use of aprotonin. Results are shown in **Figure 5**. When supplements were added, the strain significantly (*P* < 0.01) decreased threefold from 15.2 ± 1.7% to 5.1 ± 0.5% in the presence of aprotinin (**Figure 5A**). Omitting the aprotinin did not significantly affect the strain. Young’s modulus increased twofold, from 63.7 ± 4 kPa to 124.3 ± 3.2 kPa when supplements were added in the presence of aprotinin. When aprotinin was stopped, Young’s modulus significantly increased from 50.9 ± 7.0 kPa in the absence of supplements to 129.3 ± 16.6 kPa in the presence of supplements (*P* < 0.05). Independently of supplement presence (either present or absent), the omission of aprotinin did not affect Young’s modulus.

### Decrease of Fibrin Content

**FIGURE 6:**
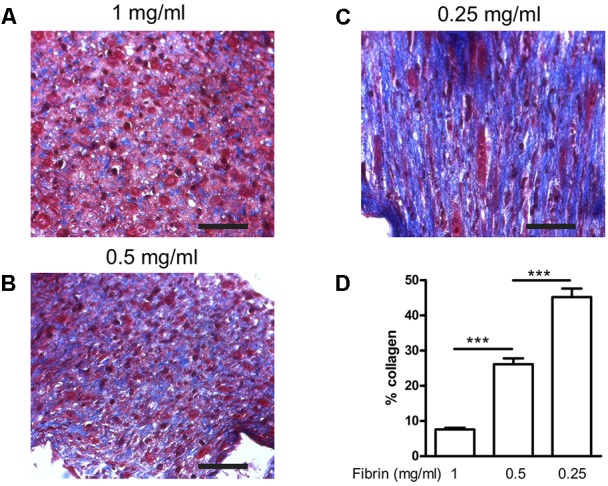
Collagen deposition induced by medium supplementation with ascorbic acid, proline, and hydroxyproline in function of decreasing fibrin concentrations. BAMs were engineered with **(A)** 1 mg/ml fibrin, **(B)** 0.5 mg/ml fibrin, **(C)** 0.25 mg/ml fibrin. Images from Trichome stained sections from different BAM groups; *blue areas* are collagen staining. Scale bar is 100 μm. **(D)** Collagen quantification depicted as the percentage of *blue area* compared to the total area of each image. ^∗∗∗^*P* < 0.001.

**FIGURE 7:**
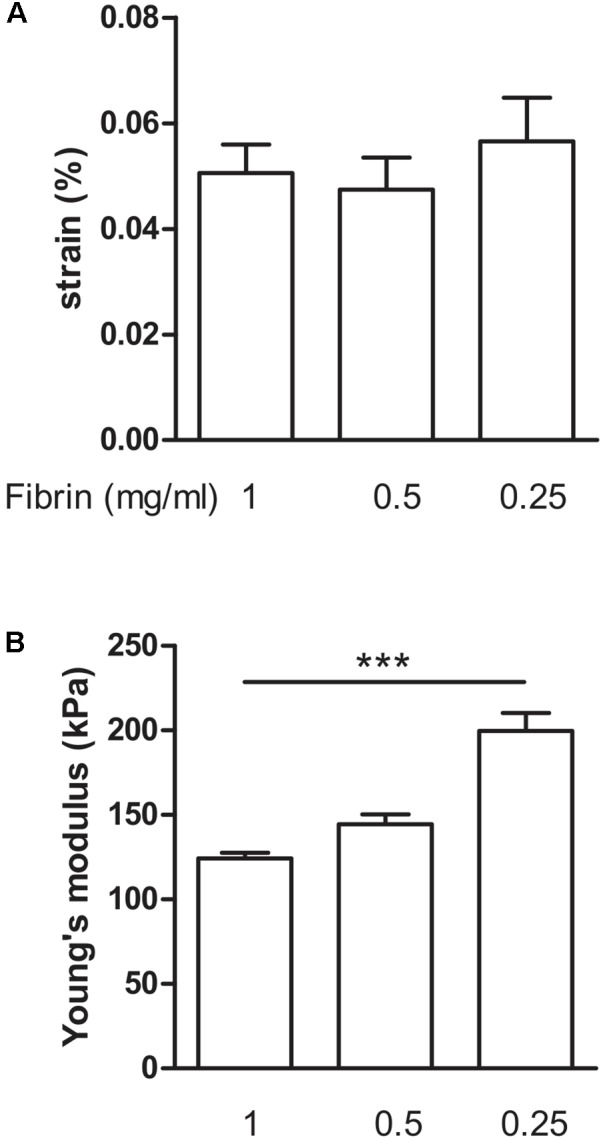
Visco-elastic effects of decreasing fibrin concentration. **(A)** Strain of the BAM at 2,500 μN force. All groups received media supplements and aprotinin. Decreasing fibrin content did not significantly affect the strain to reach a force of 2,500 μN. **(B)** Young’s modulus. Decreasing fibrin content from 1 to 0.25 mg/ml significantly increased Young’s modulus. Data are presented as mean ± SEM. ^∗∗∗^*P* < 0.001.

Next, we determined how much the initial fibrin content affects the collagen deposition, by reducing it to 0.5 and 0.25 mg/ml, in the presence of supplements. The collagen content further significantly (*P* < 0.001) increased to 26.1 ± 1.7% and 45.2 ± 2.4%, respectively (**Figure 6**). Decreasing fibrin content did not significantly affect the strain when force was applied (**Figure 7A**). Also Young’s modulus did not significantly change when decreasing to 0.5 mg/ml fibrin, but significantly increased (*P* < 0.001) from 124.3 ± 3.2 kPa to 199.8 ± 10.5 kPa when fibrin content was further decreased to 0.25 mg/ml (**Figure 7B**). This increase in Young’s modulus with decreasing fibrin content was due to the smaller cross-sectional area of the BAM. The cross-sectional area decreased from 0.51 ± 0.17 mm^2^ (1 mg/ml) to 0.28 ± 0.05 mm^2^ (0.25 mg/ml).

### Effects of Cell Number and Fibrin Cross-Linking

**FIGURE 8:**
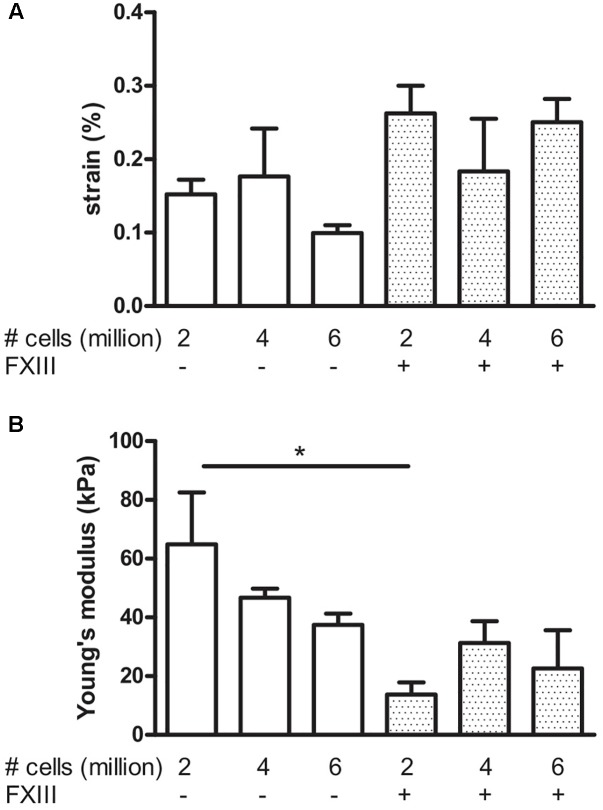
Visco-elastic effects of cell number and fibrin cross-linking. Data were acquired after 7 days culture. BAMs were engineered with 2, 4, or 6 million cells in the absence (–) or presence (+) of FXIII as indicated on the graphs. All groups received medium supplements. **(A)** Strain of the BAM at 2,500 μN force. Increasing the cell number did not significantly affect the strain to reach a force of 2,500 μN, both in presence or absence of FXIII. **(B)** Young’s modulus. Addition of FXIII significantly decreased Young’s modulus. Data are presented as mean ± SEM. ^∗^*P* < 0.05.

To determine if we could further improve the mechanical strength of the BAMs, we examined two strategies: An increase in cell number and the cross-linking of fibrin. Fibrin can be crosslinked with factor XIII (FXIII), which is a transglutaminase. Effects of these modifications, measured at 7 days post casting are shown in **Figure 8**. The effect of FXIII addition was a significant (*P* < 0.05) decrease in Young’s modulus from 64.9 ± 17.6 kPa to 13.7 ± 4.2 kPa when using 2 million cells. Increasing the cell number to 4 or 6 million cells did not significantly change the strain nor Young’s modulus, independent of FXIII addition.

### Contractile Properties of BAMs

**FIGURE 9:**
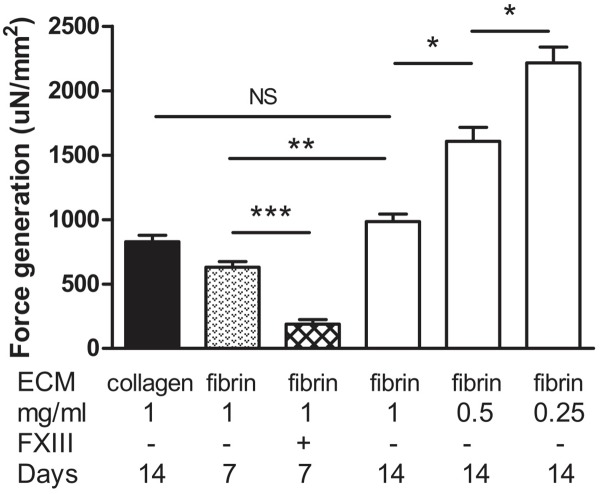
Generation of force upon tetanic stimulation of BAMs generated with a fibrin matrix with media supplementation of ascorbic acid, proline, and hydroxyproline. Force was normalized to cross-sectional area. Collagen BAMs were used as a control. Data were acquired at either 7 or 14 days after casting the BAM, as indicated. Aprotinin was present in all fibrin BAM media. Data are presented as mean ± SEM. ^∗^*P* < 0.05, ^∗∗^*P* < 0.01, and ^∗∗∗^*P* < 0.001.

To assess muscle functionality, we determined contractile force generated by electrical stimulation. The stimulation parameters to generate maximal tetanic force had been determined previously for this system (Lee and Vandenburgh, 2013). Field stimulation was performed with two platinum electrodes 4 mm apart on either side of the BAM. Maximum isometric tetanic force was obtained by applying an electrical stimulus of 40 V at 40 Hz with 4 ms wide pulses. Absolute force exerted by the BAM was normalized for cross-sectional area. As a reference, we used BAMs engineered with Zyderm. These BAMs were able to generate 829 ± 49 μN/mm^2^ force at 14 days post casting (**Figure 9**). The BAMs engineered with 1 mg/ml fibrin in the presence of media supplements did not differ significantly (985 ± 58 μN/mm^2^) (**Figure 9**). Force was lower when culture time decreased to 7 days (630 ± 90 μN/mm^2^). When FXIII had been added, this force was even lower: 189 ± 64 μN/ mm^2^. A similar absolute force was generated at 14 days by BAMs engineered with a lower fibrin content, however, due to the decreased cross-sectional area, the normalized force increased to 1,608 ± 107 μN/mm^2^ for 0.5 mg/ml fibrin and 2,215 ± 123 μN/mm^2^ for 0.25 mg/ml fibrin (**Figure 9**).

## Discussion

Bio-artificial muscle composed of aligned human skeletal myofibers, have been successfully engineered *in vitro* and have potential applications in regenerative medicine and gene therapy. BAMs in most of our previous work have been engineered scaffold-free in a hydrogel since using a synthetic PLG scaffold negatively affected myofiber alignment and degradation products were observed *in vivo* (Thorrez et al., 2008). However, when a clinically approved collagen type I (Zyderm) ECM was used for *in vivo* implantation, these BAMs did not get vascularized, leading to an impaired cell survival (Thorrez et al., 2006). This was not entirely surprising, since Zyderm is clinically used as a bulking agent for reconstructive surgery so the collagen I is processed to minimize host interaction. Therefore, we investigated the use of an alternative ECM composition for BAM engineering. Fibrin (Tisseel) is FDA-approved and widely used in the clinic as a tissue glue. Studies have shown that Tisseel fibrin can be vascularized *in vivo* (Cesarman-Maus and Hajjar, 2005).

Here, we demonstrate *in vitro* that fibrin as an ECM allows for angiogenic sprouting, but addition of collagen I (Zyderm) completely abolishes this effect (**Figure 2**). These results are in agreement with the *in vivo* anti-angiogenic nature of Zyderm as observed previously (Thorrez et al., 2006). Addition of HGF and VEGF was previously shown to greatly increase sprout formation (Matsumoto et al., 2007). With the fibrin ECM we indeed observed this effect, but even in the presence of these factors the anti-angiogenic effect of Zyderm at a relevant concentration (1 mg/ml) could not be overcome.

However, when applied as a tissue glue, Tisseel is reabsorbed during the wound healing process in 10–14 days (Spotnitz, 2014) and when mixed with myoblasts and injected intramuscularly, the fibrin matrix was completely dissolved after 2 weeks (Beier et al., 2006). Therefore, either fibrinolysis *in vivo* needs to be inhibited or autologous ECM deposition from the BAM needs to be stimulated to (partially) replace the degraded fibrin. We investigated the latter option by stimulating collagen deposition. Ascorbic acid has already been known for a long time to stimulate collagen deposition (Schwarz et al., 1981). It has been used to stimulate collagen deposition from human tenocytes for tendon tissue engineering (Hakimi et al., 2014), from vascular interstitial cells for heart valve tissue engineering (Wu et al., 2017) and from fibroblasts for vascular graft tissue engineering (Patel et al., 2018). The use of ascorbic acid-releasing scaffolds has also been suggested for use in pelvic floor repair (Mangır et al., 2016). The triple helical structure of collagen arises from the abundance of three amino acids: Glycine, proline, and hydroxyproline. Proline and hydroxyproline residues permit the sharp twisting of the collagen helix and play key roles in collagen stability (Jenkins et al., 2003). So, in addition to ascorbic acid, we also supplemented the media with hydroxyproline and proline. The primary human skeletal muscle myogenic cell isolates used in this study contained about 30% fibroblasts. It is known that fibroblasts secrete collagen and other important ECM components (Schwartz et al., 1979).

Fibrin BAMs were successfully engineered *in vitro*. **Figures 3A–C** displays the contraction of the cell-ECM mix during *in vitro* culture. After 4 days in differentiation medium, myofibers expressing tropomyosin (**Figure 3D**) had formed that aligned along the axis of the attachment sites. The fibrin BAMs were cultured in the absence or presence of the media supplements for 14 days, and we determined collagen content after 14 days *in vitro*. Addition of proline, hydroxyproline, and ascorbic acid significantly enhanced *de novo* collagen deposition in the BAMs from 4.7 to 7.6%.

Fibrin degradation can be controlled by modulating the fibrinolysis. Aprotinin is required in media during the first days in culture after the BAM engineering to prevent fibrinolysis. Allowing fibrinolysis by stopping aprotinin addition at day 5 to simulate what happens after *in vivo* implantation of the BAMs had no significant effect on the BAMs when no media supplements were present. In the presence of supplements, no significant effect was observed on Young’s modulus or strain at 2,500 μN, but when fibrinolysis was allowed to take place a significantly greater amount of the ECM was composed of collagen.

The supplement effect was present both in the presence or absence of fibrinolysis. However, by stopping aprotinin addition and thus allowing for fibrinolysis, we observed a further increase in collagen production to 13.6% (**Figure 4**). This is consistent with a previous report showing that vascular smooth muscle cells cultured in fibrin increased their collagen deposition due to decreasing concentrations of the fibrinolysis inhibitor 𝜀-aminocaproic acid (Ahmann et al., 2010).

BAM mechanical strength was evaluated by visco-elastic testing. BAMs to which the media supplements proline, hydroxyproline, and ascorbic acid were added displayed a significantly higher Young’s modulus than BAMs which lacked these media supplements. This effect was demonstrated both in the presence and the absence (stopped at day 5) of aprotinin. Addition of proline, hydroxyproline, and ascorbic acid increased the stiffness of the BAMs, allowing to resist 2,500 μN force at lower strain. Therefore, the addition of proline, hydroxyproline, and ascorbic acid enhances the amount of collagen *de novo* deposited by the cells in the fibrin BAMs and renders them more resistant to strain by externally applied force.

Collagen deposition and visco-elastic properties of the BAMs changed with varying fibrinogen content. When a lower concentration of fibrinogen was used for engineering the BAMs, more collagen was deposited, up to 45.2%. Moreover, a higher Young’s modulus was measured, and BAMs were able to still respond to 2,500 μN of force with a low strain.

Bonds formed by transglutaminase exhibit a higher resistance to proteolysis. Therefore, we used FXIII, a transglutaminase which cross-links fibrin in the BAM engineering process. FXIII resulted in a slight increase of strain at 2,500 μN applied force and a lower Young’s modulus. Also, we varied the number of cells per BAM. The cell number did not significantly change the visco-elastic parameters of the BAM.

The elastic moduli of native murine muscle have been measured by others with the use of AFM (Engler et al., 2004). Results varied from a Young’s modulus of an extensor digitorum longus muscle of 12 ± 4 kPa (Engler et al., 2004) to 61 ± 5 kPa for single fibers of the flexor digitorum brevis muscle (Defranchi et al., 2005). AFM measurements on monolayer murine myoblasts yielded a Young’s modulus of 11.5 ± 1.3 kPa for undifferentiated myoblasts, increasing to 45.3 ± 4.0 kPa after 8 days of differentiation (Collinsworth et al., 2002). This increase is consistent with our data; we observed a Young’s modulus in the range of 40–60 kPa 7 days after casting (**Figure 8**), increasing to a range of 50–200 kPa measured at 14 days after casting (**Figures 5**, **7**). However, AFM measurement of the elastic modulus differs from our measurement method. First, AFM is based on measurements with a small cantilever, which registers local forces on a cellular scale rather than a whole-tissue scale, whereas our method measured forces generated by the entire tissue. Second, the AFM cantilever registers forces perpendicular (transversal) to the alignment of the myofibers, whereas tensile testing measures forces in the longitudinal direction, similar to the direction in which a muscle exerts its function. Therefore, we believe that tensile testing provides information which is physiologically more relevant than AFM measurements. Tensile testing of tissue engineered skeletal muscle has been reported to yield a Young’s modulus in the range of 12–31 kPa (Heher et al., 2015). However, in the latter study, a murine cell line was used, fibrin densities were much higher (10–40 mg/ml) and the fusion index (15–30%) was lower than what we obtain with human cells (70%) (Gholobova et al., 2015).

Important for muscle physiology is the amount of force that can be generated by the myofibers upon electrical stimulation. Fibrin (1 mg/ml) BAMs, cultured in the presence of media supplements, were able to generate 985 ± 58 μN/mm^2^ force at 14 days after casting. This was similar to collagen BAMs (829 ± 49 μN/mm^2^). However, the fibrin BAMs at 14 days had a larger cross-sectional area, so the absolute force generation of the BAMs was higher. When decreasing the fibrin content, this absolute force remained similar, but due to decreased cross-sectional area, the normalized force increased, up to 2,215 ± 123 μN/mm^2^ for 0.25 mg/ml fibrin. Cross-linking of fibrin with FXIII significantly impeded force generation (189 ± 64 μN/mm^2^). The forces we report here are in the same range as what we and others previously reported (Huang et al., 2005; Brady et al., 2008; Vandenburgh et al., 2008; Fuoco et al., 2015; Madden et al., 2015; Martin et al., 2017; Kasper et al., 2018). Forces exerted by excised tibialis anterior muscle were around 1–2 mN (Cook et al., 2016), thus the forces we obtain are only slightly lower. A longer culture time *in vitro* may further enhance contractile properties (Lee and Vandenburgh, 2013).

Other methods to increase collagen deposition may be utilized in parallel to or instead of stimulation by ascorbic acid. For example, addition of fibrin degradation products (Ahmann et al., 2010) and electrical stimulation (Rahimi et al., 2014) have been shown to enhance collagen deposition. Furthermore, ascorbic acid has a toxic effect at higher concentration, but magnesium ascorbyl phosphate, a stable derivative of ascorbic acid, was shown to be usable at higher concentrations with human tenocytes (Hakimi et al., 2014).

A limitation of the current study is that BAMs were cultured under static conditions. Mechanical conditioning of BAMs has been reported to maintain a constant elastic modulus over 8 days of stretching, whereas control BAMs become stiffer (Powell C.A. et al., 2002). Another future challenge is to study the effect of the collagen deposition *in vivo*. Importantly, it remains to be seen if the deposited collagen does allow angiogenesis in the BAM. The lack of angiogenesis in the BAM which we observed previously (Thorrez et al., 2006) may also be partly due to the lack of tension of the myofibers after implantation. This tension may be needed for proper myofiber morphology and vascularization. New ways of implanting BAMs under tension need to be developed. Alternatively, inclusion of certain growth factors may facilitate the vascularization process (Thorrez et al., 2006). Neurotrophic (Iwakawa et al., 2001) and angiogenic (Wong et al., 2003) factors can be incorporated in fibrin by simply suspending the factor in the hydrogel. Most likely a combination of these growth factors will be needed; indeed inclusion of VEGF_165_ with bFGF or VEGF_121_ was shown to induce more mature blood vessels than the individual growth factors (Wong et al., 2003). Bioactive peptides can also be enzymatically linked to the fibrin matrix (Schense et al., 2000).

## Conclusion

In conclusion, contracted, differentiated BAMs can be successfully engineered in a fibrin ECM. The addition of proline, hydroxyproline, and ascorbic acid to the media, together with decreasing the fibrinogen content, increases the visco-elastic properties of the BAMs as well as the amount of *de novo* collagen ECM deposited. This physico-chemical modulation of the ECM may be beneficial for future *in vivo* applications by maintaining BAM consistency and survival while allowing angiogenesis.

## Author Contributions

LT and HV contributed conception and designed the experiments. LT, JS, and KD performed the experiments and statistical analysis. LT and KD wrote the first draft of the manuscript. HV and LT acquired funding for the work. LT, HV, JS, and KD wrote sections of the manuscript.

## Conflict of Interest Statement

The authors declare that the research was conducted in the absence of any commercial or financial relationships that could be construed as a potential conflict of interest.

## Abbreviations

AFM: atomic force microscopy
BAM: bio-artificial muscle
bFGF: basic fibroblast growth factor
DAB: 3,3′-diaminobenzidene
EBM-2: endothelial cell basal medium-2
ECM: extracellular matrix
FBS: fetal bovine serum
HGF: hepatocyte growth factor
HUVEC: human umbilical vein endothelial cell
PLG: poly-lactide-co-glycolide
SEM: standard error of the mean
SkGM: skeletal muscle growth medium
VEGF: vascular endothelial growth factor
